# Qiliqiangxin inhibits angiotensin II-induced transdifferentiation of rat cardiac fibroblasts through suppressing interleukin-6

**DOI:** 10.1111/jcmm.12512

**Published:** 2015-03-06

**Authors:** Jingmin Zhou, Kun Jiang, Xuefeng Ding, Mingqiang Fu, Shijun Wang, Lingti Zhu, Tao He, Jingfeng Wang, Aijun Sun, Kai Hu, Li Chen, Yunzeng Zou, Junbo Ge

**Affiliations:** a Department of Cardiology, Shanghai Institute of Cardiovascular Diseases, Zhongshan Hospital, Fudan UniversityShanghai, China; b North Sichuan Medical College, Department of Cardiology, The Affiliated Hospital of North Sichuan Medical CollegeNanchong, Sichuan, China

**Keywords:** qiliqiangxin, cardiac fibroblast, interleukin-6, transdifferentiation, NFAT3

## Abstract

Qiliqiangxin (QL), a traditional Chinese medicine, had long been used to treat chronic heart failure. Recent studies revealed that differentiation of cardiac fibroblasts (CFs) into myofibroblasts played an important role in cardiac remodelling and development of heart failure, however, little was known about the underlying mechanism and whether QL treatment being involved. This study aimed to investigate the effects of QL on angiotensin II (AngII)-induced CFs transdifferentiation. Study was performed on *in vitro* cultured CFs from Sprague–Dawley rats. CFs differentiation was induced by AngII, which was attenuated by QL through reducing transforming growth factor-β_1_ (TGF-β_1_) and α-smooth muscle actin (α-SMA). Our data showed that AngII-induced IL-6 mRNA as well as typeI and typeIII collagens were reduced by QL. IL-6 deficiency could suppress TGF-β_1_ and α-SMA, and both IL-6 siRNA and QL-mediated such effect was reversed by foresed expression of recombined IL-6. Increase in actin stress fibres reflected the process of CFs differentiation, we found stress fibres were enhanced after AngII stimulation, which was attenuated by pre-treating CFs with QL or IL-6 siRNA, and re-enhanced after rIL-6 treatment. Importantly, we showed that calcineurin-dependent NFAT3 nuclear translocation was essential to AngII-mediated IL-6 transcription, QL mimicked the effect of FK506, the calcineurin inhibitor, on suppression of IL-6 expression and stress fibres formation. Collectively, our data demonstrated the negative regulation of CFs differentiation by QL through an IL-6 transcriptional mechanism that depends on inhibition of calcineurin/NFAT3 signalling.

## Introduction

Myocardial fibrosis is a multi-factorial process which includes the progressively increased transdifferentiation of cardiac fibroblasts (CFs) into myofibroblasts. The phenotype of ‘persistent myofibroblast’ may lead to increased production of extracellular matrix (ECM), including collagen deposition, enhanced expresson and translocation of α-smooth muscle actin (α-SMA) and transforming growth factor-β_1_ (TGF-β_1_) signalling activation. These events are essential for the development of cardiac remodelling and chronic heart failure 1–3.

Qiliqiangxin (QL) is a traditional Chinese herb medicine, including mainly Radix Astragali, Radix Ginseng, Salvia Miltiorrhiza, *etc*. Recent studies show that QL could improve both systolic and diastolic cardiac function in spontaneously hypertensive rats through inhibiting cardiac chymase-mediated angiotensin II production 4. Pressure overload-induced myocardial hypertrophy and inflammation response is alleviated by QL through inhibiting the ErbB receptors signalling and CBP/p300 nuclear transaction 5. QL also has effect on balancing the pro-inflammatory and anti-inflammatory cytokines during myocardial infarction 6. Recently, QL is proved to be effective on inhibition of calcium signalling by blocking the L-type Ca^2+^ channel (*I*_*Ca-L*_), which weakens myocardial contractility and improves cardiac remodelling 7. However, the protective role of QL in heart, particularly which kind of cardiac cell is involved, is not well understood. CFs are the primary cardiac cells present in myocardium and participate in the formation of the myocardial ECM. Transdifferentiation of CFs into myofibroblast plays a critical role in fibrillar collagens deposition and cardiac fibrosis 8,9. Therefore, we test the hypothesis that QL might improve cardiac function through regulating CFs transdifferentiation.

Recent studies demonstrated that Angiotensin II (AngII) plays a important role in fibroblasts differentiation 10,11, and AngII is also critically involved in chronic inflammation and progessive fibrosis during pressure overload 12–14. AngII infusion significantly increased Interleukin-6(IL-6) expression in rats’ heart, meanwhile elevated IL-6 serum levels promoted cardiac fibrosis through TGF-β_1_/Smad (TGF-β_1_/Smad) signalling pathway 15, and promoting transdifferentiation of CFs into myofibroblasts 16,17. Myofibroblasts formation promotes the profibrotic response and cardiac remodelling 18 in pressure overload hearts 5 and cardiomyopathy 19. Meanwhile, evidence show that IL-6 significantly increases collagen deposition and ventricular stiffness 17. Anti-inflammatory therapy might be a promising strategy against CFs transdifferentiation and cardiac fibrosis 20. Regarding to the anti-inflammatory role of QL in pressure overloaded heart, in this study we investigate the effect of QL on AngII-induced CFs, the possible mechanism for QL on CFs transdifferentiation and inflammation targets.

## Materials and methods

### Materials

Low-glucose DMEM, foetal bovine serum (FBS), Trypsin and HBSS were from Gibco Pasadena, CA (USA). Qiliqiangxin extracts were provided by Shijiazhuang Yiling Pharmaceutical Co., Shijiazhuang Ltd (China); Olmesartan (OLM) was purchased from Shanghai Sankyo Pharmaceutical Co., Shanghai Ltd. (China); Ang II was purchased from Sigma-Aldrich Co., St. Louis, Missouri LLC. (USA); Rabbit anti-rat α-SMA polyclonal antibody was from Bioworld Technology, Nanjing Inc. (China); Rabbit anti-rat TGF-β_1_ polyclonal antibody was from Santa Cruz Dallas, Texas (USA). Rat IL-6 Quantikine ELISA Kit was from R&D Systems, Minneapolis, MN Inc (USA), IL-6 RNA lentivirus vector was from Shanghai GeneChem Co., Shanghai Ltd (China).

### Cardiac fibroblasts isolation and treatment

1–3-day-old neonatal Sprague-Dawley rats were purchased from Shanghai Slac Laboratory Animal Co., Ltd. Cardiac fibroblasts were isolated, as described previously 21, briefly, trypsin digestion and differential attachment, and adherent cells were cultured in low-glucose DMEM supplemented with 10% FBS and 1% penicillin-streptomycin in a humidified incubator with 5% CO_2_ and 95% air. The CFs of passage 2 were 40–50% confluent, and a portion of the cells transfected with IL-6 small-interfering RNA (IL-6-siRNA with green fluorescence protein labelled) at a multiplicity of infection of 10 and 48 hrs after transfection, CFs were harvested for further analysis. After the cells were 80–90% confluent, the medium was replaced by serum-free DMEM the day before pre-treated with QL (effective intervention dose, 0.5 mg/ml) or OLM (10^−7^ mol/l) for 30 min. and exposed to AngII (100 nM) for 24 hrs.

### Real time polymerase chain reaction

Total RNA was isolated from the CFs using TRIzol reagent (Invitrogen Carlsbad, CA, USA). Primer for IL-6 forward 5′-GCC AGA GTC ATT CAG AGC AAT A-3′ and reverse: 5′-TTA GGA GAG CAT TGG AAG TTG G-3′, for Collagen-I forward 5′-CCA ATG GTG CTC CTG GTA TT-3′ and reverse: 5′-GTT CAC CAC TGT TGC CTT TG-3′, for Collagen III forward 5′-AAT GGC TCT CCA GGA CAA AG-3′ and reverse: 5′-CCA CCA GGA CTG CCA TTA TT-3′, reverse: 55′-GTT CAC CAC TGT TGC CTT TG-3′ and for GAPDH forward 5′-ACT CCC ATT CTT CCA CCT TTG-3′ and reverse: 5′-CCC TGT TGC TGT AGC CAT ATT-3′, the mRNA levels of IL-6, Collagen-I and -III were normalized to GADPH. PCR cycling conditions: 95°C 60 sec., 95°C 15 sec., PCR cycles (×40 cycles): 95°C 15 sec., 65°C 25 sec. All data analyses were used 2−ΔCt method.

### Western blot analysis

The proteins in CFs were lysed using RIPA (radioimmunoprecipitation) lysis buffer supplemented with 1 mmol/l PMSF and phosphates inhibitor (Pierce, Rockford, IL, USA). For immunoblotting:equal amounts of proteins were resolved on 12% SDS-polyacrylamide gel and transferred onto poly-vinylidene fluoride membrane (Thermo Scientific), Andingmen East Street, Beijing, China After blocked with 5% bovine serum albumin (BSA) at room temperature for 1 hr, Membranes were exposed to primary antibody anti-GAPDH (1:2000 diluted in TBST), anti-a-SMA (1:1000 dilution), anti-TGF-β_1_ (1:1000 dilution), over night at 4°C and incubated with HRP-conjugated secondary antibody for 2 hrs at room temperature. The signal was detected with the ECL Plus (Thermo Scientific). Bands were analysed by densitometry using Quantity One software (Hercules, CA, USA).

### Immunofluorescence quantification and analyses

The expression levels of α-SMA were also detected by immunofluorescence staining. CFs were treatment with QL (0.5 mg/ml) or OLM (10 nM), and/or stimulated by Ang II (100 nmol/l) for 24 hrs, the cells were washed by PBS, fixed with 4% paraformaldehyde and permeabilized with 0.1% Triton X-100. After the cells were incubated with BSA for 30 min., the primary antibody specific for α-SMA was added overnight at 4°C. And the cells were incubated with Fluorescein isothiocyanat (FITC) secondary antibody for 1 hr at room temperature in the dark room, and then DAPI was added for 30 min. The cells were visualized under fluorescence microscope.

Fluorescence intensity of cells was examined and quantified by the specialized measurement incorporated within imaging software. Fluorescence intensities could be compared between regions of the same set of stress fibres and between cells of different treatments. The data were analysed by the mean fluorescence intensities from 10 random selected regions, each with a standard 50-μm^2^ circular region.

### Statistics

All data were expressed as means ± SEM. The statistical significance was assessed by using one-way anova for multiple-group comparisons. Differences between groups were using paired-sample Student's *t*-test, *P* < 0.05 was considered statistically significant.

## Results

### QL effectively reversed AngII-induced CFs transdifferentiation

**Figure 1:**
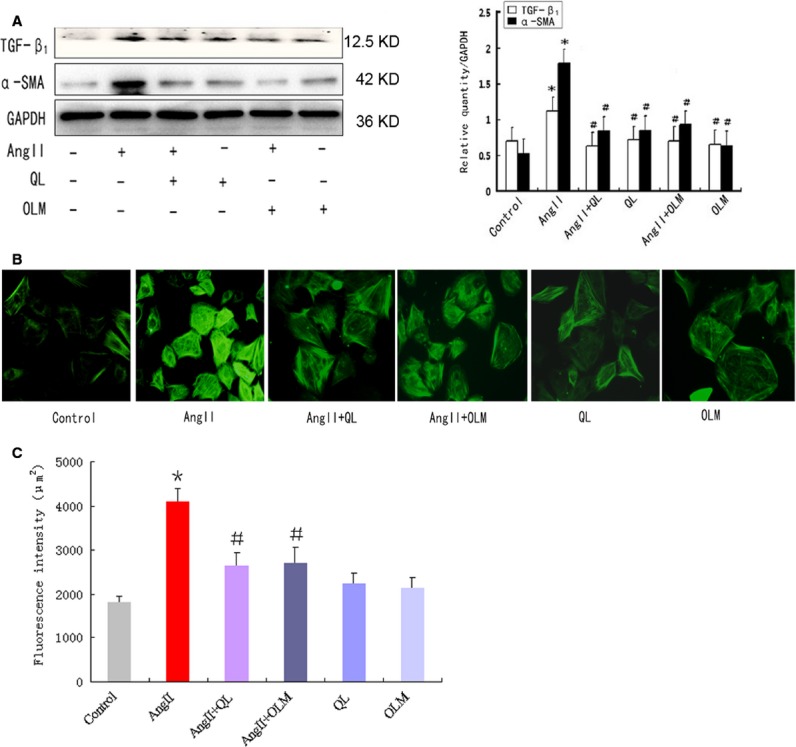
QL effectively reversed AngII-mediated CFs transdiferentiation. CFs were stimulated by AngII (100 nmol/l) for 24 hrs with or without the pre-treatment of QL (0.5 mg/ml) or Olmesartan (10^−7^ mol/l). (A) The protein levels of TGF-β_1_ and α-SMA were detected by Western blot. (B) Actin stress fibres expressed in CFs was detected by fluorescent labelling with FITC-conjugated antibody against α-SMA. (C) Quantification of mean fluorescence intensity of stress fibres in CFs with α-SMA staining. *indicates *P* < 0.05 *versus* Control group; ^#^indicates *P* < 0.05 *versus* AngII-induced group.

To investigate the role of QL in regulating CFs transdifferentiation, we first induced the *in vitro* cultured CFs by AngII (100 nmol/l), which played an important role in CFs transdifferentiation and the process of epithelial mesenchymal transition. After treatment for 24 hrs, the stage-specific transdifferentiation markers, TGF-β_1_ and α-SMA, were detected by Western blot. As shown in Figure1A, AngII-induced TGF-β_1_ and α-SMA in CFs were significantly reduced by pre-treatment with QL (0.5 mg/ml) or OLM (10 nM). The optimized concentration of QL was determined by the effects of concentration gradient on CFs viability and their inhibition of α-SMA (Figs S2 and S3). However, the increases of TGF-β_1_ and α-SMA were not affected by QL or OLM without the induction of AngII.

Besides the increase in total level of α-SMA, the subcellular localization of α-SMA attached much more importance of functional myofibroblasts that transdifferentiated from CFs. α-SMA was incorporated into actin stress fibres to enhance cell contractility 22. Thus, we next quantified the stress fibres in CFs by immunofluorescence staining with anti-α-SMA (Fig.1B). AngII stimulation for 48 hrs led to enhancement of actin stress fibres, which was diminished by pre-treatment with QL or OLM (Fig.1C), indicating that QL might prevent α-SMA recruitment to stress fibres through inhibition of AngII signalling.

### QL reversed AngII-induced CFs transdifferentiation through inhibiting IL-6

**Figure 2:**
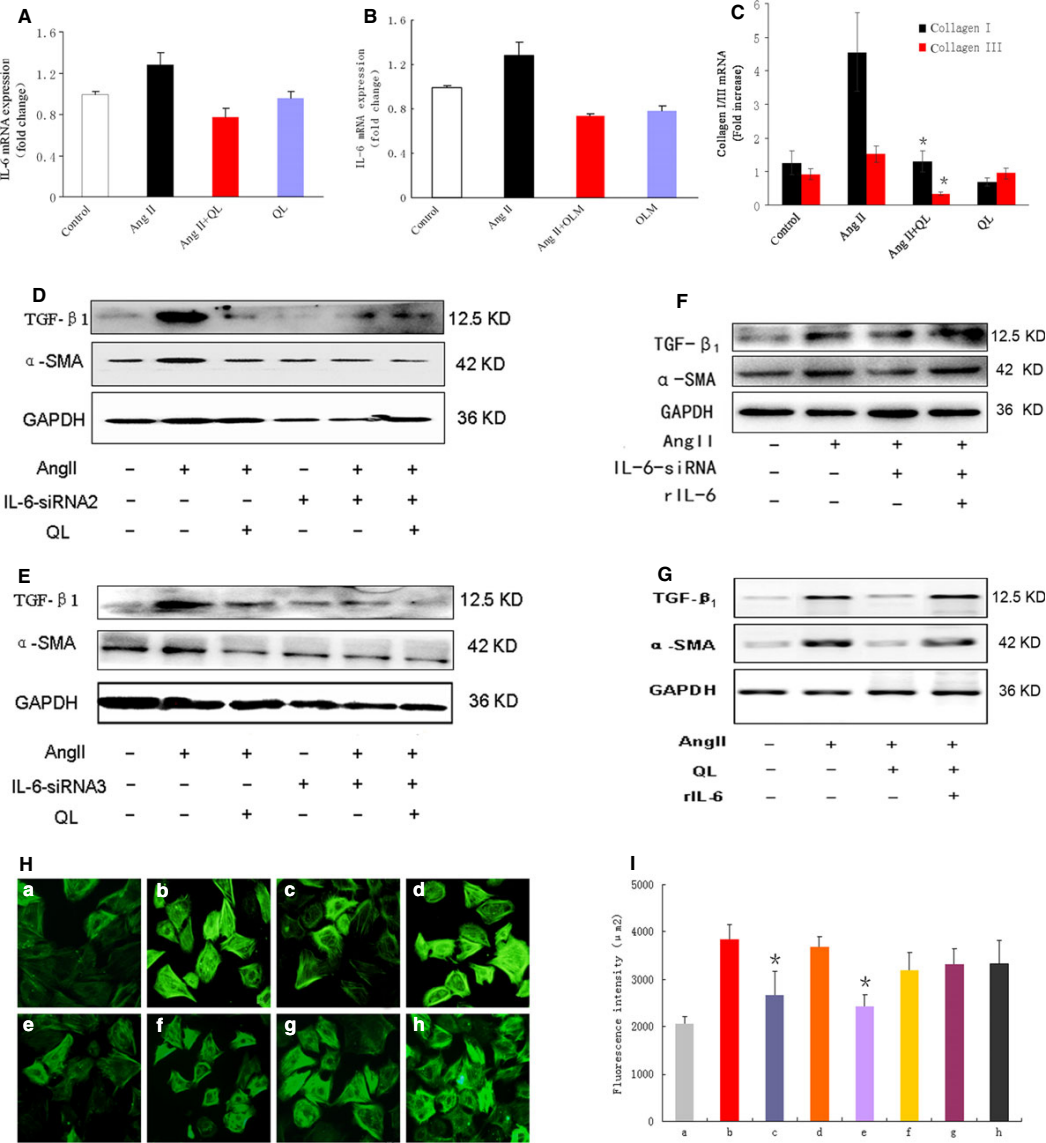
QL reversed AngII-mediated CFs transdiffentation *via* inhibiting IL-6 transcription. IL-6 in CFs were silenced by small-interfering RNA (siRNA) lentivirus transfection for 48 hrs and stimulated with AngII for another 24 hrs. (A) IL-6 mRNA expression was detected by real time polymerase chain reaction (RT-PCR) in CFs in the presence or absence of QL or (B) OLM. (C) The mRNA levels of Collagen type I and type III in CFs pre-treated with or without QL. (D and E) The effect of QL on AngII-mediated TGF-β_1_ and α-SMA expressions in the presence or absence of two different kinds of IL-6 siRNA. (F and G) The effect of rIL-6 (20 ng/ml) on QL or IL-6 siRNA-induced suppression of TGF-β_1_ and α-SMA. (H) Actin stress fibres was detected by α-SMA staining in CFs from different treated groups (a, control; b, AngII; c, AngII+IL-6 siRNA; d, AngII+ control siRNA; e, AngII+QL; f, AngII+QL+rIL-6; g, AngII+IL-6 siRNA+rIL-6; h, rIL-6). (I) Quantification of mean fluorescence intensity of stress fibres in CFs with α-SMA staining. *indicates *P* < 0.05 *versus* AngII-induced group.

Previous studies revealed that IL-6 played an important role in fibroblasts transdifferentiation 12, therefore, we tested the possibility whether IL-6 was regulated by QL. First, our data showed that the transcriptional level of IL-6 was enhanced in CFs by AngII stimulation, and significantly reduced by pre-treating cells with QL or OLM, respectively (Fig.2A and B), suggesting that IL-6 was activated by AngII signalling and could be down-regulated by QL. Importantly, the enhancement of type I and type III collagens in CFs by AngII stimulation for 24 hrs was significantly reversed by QL (Fig.2C), indicating a critical role of QL in reversing CFs transdifferentiation.

To confirm the role of IL-6 in CFs transdifferentiation, we knocked down IL-6 by transfection of small-interfering RNA (siRNA) in CFs. We tested the effects of three pairs of siRNA on IL-6 inhibition, the siRNA sequences were shown in Table1. SiRNA1 failed to inhibit IL-6 expression, but both siRNA2 and siRNA3 effectively down-regulated the protein level of IL-6 (Fig. S1). Thus, we chose these two siRNAs against IL-6 in the following experiments.

**Table 1:** The list of the designed siRNAs against IL-6

IL-6	siRNA sequence			
siRNA1	S 5′:		GCUAUGCCUAAGCAUAUCA	UU
	mRNA:	GT	GCTATGCCTAAGCATATCA	GT
	AS 3′:	UU	CGAUACGGAUUCGUAUAGU	
siRNA2	S 5′:		CAGACCAGUAUAUACCACU	UU
	mRNA:	AA	CAGACCAGTATATACCACT	TC
	AS 3′:	UU	GUCUGGUCAUAUAUGGUGA	
siRNA3	S 5′:		CUAAAGGUCACUAUGAGGU	UU
	mRNA:	TT	CTAAAGGTCACTATGAGGT	CT
	AS 3′:	UU	GAUUUCCAGUGAUACUCCA	

Next, we further investigate the key role of IL-6 in inducing CFs transdifferentiation, we determined the expression of TGF-β_1_ and α-SMA induced by AngII in IL-6 knockingdown CFs. As shown in Figure2, both siRNA2 and siRNA3 against IL-6 could mimic the effect of QL in inhibiting TGF-β_1_ and α-SMA, importantly OL-induced reduction in TGF-β_1_ and α-SMA was not further enhanced by adding IL-6 siRNA (Fig.2D and E), indicating that QL and IL-6 might regulate AngII-mediated CFs transdifferentiation *via* the same pathway which was dependent on IL-6 activation.

Considering that the active form of IL-6 could be secreted into the extracellular, and functioned on the adjacent cells through paracrine modes, we analysed the regain-function of IL-6 by inducing CFs with recommended IL-6 to see if restore IL-6 function that knocking down by siRNA might promote CFs transdifferentiation again. As expected, TGF-β_1_ and α-SMA were rebounded by rIL-6 treatment in either IL6-siRNA or QL stimulated CFs (Fig.2F and G). Finally, we confirmed that α-SMA staining and stress fibres quantification were significantly reduced by QL or IL-6 siRNA treatment in AngII-induced CFs, these effects were reversed by adding rIL-6 (Fig.2H and I).

### QL inhibited IL-6 through regulating nuclear NFAT3 activity

**Figure 3:**
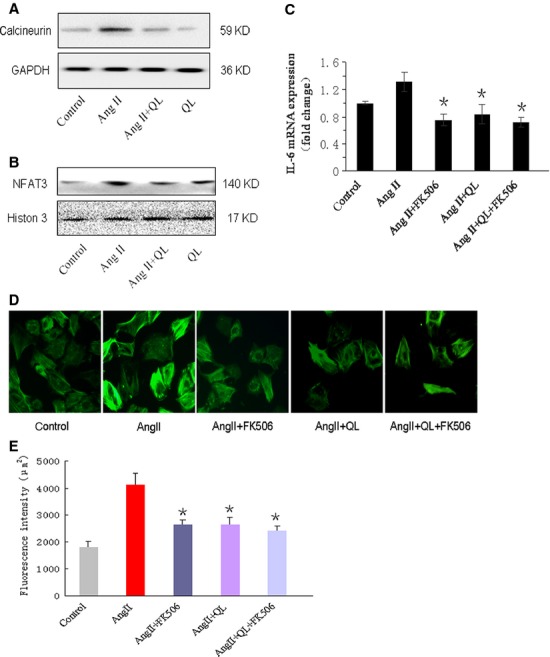
QL suppressed the transcription of IL-6 through regulating calcineurin/NFAT3 signalling pathway. CFs were stimulated by AngII (100 nmol/l) for 24 hrs with or without the pre-treatment of QL (0.5 mg/ml), (A) the cytoplasm level of calcineurin and (B) nuclear level of NFAT3 were detected by Western blot, respectively, in separated protein extracts. (C) CFs were pre-treated with or without FK506 (10^−5^ mol/l) for 1 hr before AngII stimulation, the effects of QL and FK506 on IL-6 mRNA was detected in different groups. (D) Actin stress fibres were detected by a-SMA staining in CFs from different treated groups (control, AngII, AngII with FK506, AngII with QL, AngII with FK506 and QL). (E) Quantification of mean fluorescence intensity of stress fibres in CFs with a-SMA staining. *indicates *P*< 0.05 versus AngII-induced group.

Skeletal muscle cells and CFs are the major sources of IL-6, a growing body of evidence indicated that IL-6 transcription is highly associated with the calcium signalling triggered by endured exercise. Enhanced calcineurin activity is required for the IL-6 secrition both *in vitro* and *in vivo*. Here, we found that calcineurin was increased and nuclear NFAT3 was accumulated in AngII-treated CFs, these levels were greatly reduced by QL pre-treatment (Fig.3A and 3B). To confirm whether QL affected IL-6 expression through regulating calcineurin/NFAT3 pathway, we pre-treated CFs with FK506, the calcineurin inhibitor, for 1 hr, then IL-6 mRNA expression was examined after AngII induction for 24 hrs. The results showed that NFAT3 inhibition significantly reduced IL-6 in CFs, and this effect was not further streghtened by adding QL. (Fig.3C), suggesting NFAT3 signalling might be down-regulated by QL, which led to suppression of IL-6 transcription. Furthermore, FK506 pre-treatment attenuated stress fibres in AngII-induced CFs, and there had little difference between groups with or without QL (Fig.3D and E).

## Discussion

The major finding of this study was that, (*i*) QL inhibited AngII-induced CFs transdifferentiation *via* reducing IL-6 transcription; (*ii*) NFAT3 nuclear translocation was induced by AngII, which played a critical role in IL-6 secretion and enhancement of TGF-β_1_ and α-SMA and (*iii*) QL reversed CFs transdifferentiation possibly through regulating nuclear activity of NFAT3.

AngII, the one of most important renin-angiotensin system components, played a critical role in cardiac remodelling. Recently investigations focused its impact on CFs transdifferentiation 10,23. Because, CFs conversion into myofibroblasts resulted in collagen production and deposition, which might increase the risk of myocardial fibrosis and remodelling 24. In this study, we found that AngII stimulation increased expressions of TGF-β_1_ and α-SMA, also enhanced actin stress fibres in CFs. Attenuation of these effects by QL suggested its important role in reversion of. In addition, we noted that QL had similar effect as OLM, the AT_1_R inhibitor, both of which effectively suppressed the process of CFs transdifferentiation induced by AngII, indicating that QL inhibited CFs transdifferentiation through AT_1_ receptor signalling. Considerable evidence proved that AngII-mediated AT_1_ receptor activation could transactivated TGF-β_1_ signalling under pressure overload 23,25. TGF-β_1_ signalling was essential for cardiac fibrosis, and blocking TGF-β_1_ effectively reversed CFs transdifferentiation and reduced ECM deposition, which might be the therapeutic target for myocardial fibrosis 26.

Association between inflammation response and CFs transdifferentiation was crucial for the development of cardiac fibrosis 27. Inflammatory infiltrates produce various cytokines including prototypical inflammatory cytokines such as TNF-α, IL-6 and IL-1β. Several studies proved that IL-6 played a key role in collagen deposition, fibrosis and CFs transdifferentiation 15,17,28. IL-6-mediated myofibroblasts conversion was causal for the process of cardiac fibrosis and remodelling. TGF-β_1_ and α-SMA were significantly lower or even absent in hearts of interleukin-6 knockout (IL-6^−/−^) mice than in wild-type mice after AngII infusion. Gene analysis showed that IL-6 was mainly present in CFs rather than in cardiomyocytes and macrophages after AngII administration 15. In this experiment, we also observed the expression of IL-6 in CFs was significantly increased by AngII induction. Importantly, QL effectively attenuated expression of IL-6 in AngII-stimulated CFs, and QL mimicked the effect of IL-6 siRNA on inhibition of TGF-β_1_ and α-SMA expressions, reduction in actin stress fibres, and reversion of collagen deposition. But QL did not amplify the inhibitory effect on CFs transdifferentiation in the presence of IL-6 siRNA, suggesting IL-6 activation and downstream signalling pathway was critically involved in AngII-induced TGF-β_1_ signalling and CFs transdifferentiation, QL inhibited CFs transdifferentiation, at least in part, *via* down-regulating IL-6/TGF-β_1_ signalling pathway.

AT_1_R inhibitor was postulated to have therapeutic effects on inflammatory conditions. Nishio *et al*. 29 previously demonstrated that OLM could improve the LV hypertrophy, fibrosis and diastolic dysfunction though attenuation of inflammatory response in a mouse model of diastolic heart failure. Candesartan prevented expressions of TNF-α, cyclooxygenase-2 and IL-6. The increase in inflammatory cytokines, such as IL-6, was highly associated with cardiac remodelling 30 and dilated cardiomyopathy 31. In our study, we found that IL-6 was significantly down-regulated by QL, especially knocking down of IL-6 could reverse the process of CFs transdifferentiation.

Another important finding of this study was that QL suppressed the IL-6 expression *via* negative regulation of calcineurin/NFAT3 signalling. Despite the large populations of immune cells, skeletal muscle cells and CFs might be the major sources of IL-6 production. However, the precise mechanism of CFs-mediated IL-6 activation was quite different from that mediated by macrophage or Th2 lymphocytes 32,33. A growing body of evidence indicated that IL-6 transcription is highly associated with the calcium signalling triggered by endured exercise. Enhanced calcineurin activity is required for the IL-6 secrition both *in vitro* and *in vivo* 34–36. We previously proved that AngII activated calcineurin and led to cardiac hypertrophy in a pressure overload-induced heart remodelling model 37. In this study, we found that QL effectively inhibited AngII-mediated calcineurin activation and NFAT3 nuclear translocation, which might contribute to the inhibition of IL-6 expression, because FK506 pre-treatment could reverse the transcription of IL-6. Wei *et al*. reported that L-type Ca^2+^ channel (*I*_*Ca-L*_) was blocked by QL and myocardial contractility was weakened because of reduced Ca^2+^ influx 7. Thus, we assumed that calcineurin might be the key point for QL-mediated inhibition of *I*_*Ca-L*_, which not only attenuated cardioamyocytes hypertrophy but also suppressed CFs transdifferentiation. QL-mediated reducing of Ca^2+^ influx affected localization of α-SMA at actin stress fibres, and blunted the process of conversion from CFs to myofibroblasts.

Several nuclear transcriptional factors could be activated by calcineurin, such MEF2, NFAT3 and CREB, these factors were all involved in cardiac remodelling 38–40. But here, we focused on the change in nuclear NFAT3 that suppressed by QL, NFAT3 inhibition by FK506 effectively attenuated IL-6 expression. Although there was no evidence provided that IL-6 was regulated by NFAT3, cotransfection of a constitutively active or wide-type form of NFAT3 did increase the IL-6 promoter activity. IL-6 secretion is highly associated with the calcineurin activity during muscle contraction. In our data, we proved that QL could suppress calcineurin, but till now, it was hard to prove which herb mainly contributed to the effect on *I*_*Ca-L*_ channel, our further investigation would attach the technique to separate the effective intergradient of QL capsules, and their impacts on calcium signalling, IL-6 expression and CFs transdifferentiation.

In conclusion, our present data supported a role for QL in the reversion of CFs transdifferentiation, which was achieved by suppressing the calcineurin activity, down-regulating IL-6 transcription and downstream TGF-β_1_ and α-SMA expressions. The experiment indicated a new perspective in the molecular mechanisms of QL on CFs transdifferentiation and cardiac fibrosis.

## Supporting Information

**Figure S1** Western blot analysis of the effects of different siRNAs on IL-6 inhibiton.

**Figure S2** Cell viability was determined in culured CFs stimulated by indicated concentrations of QL.

**Figure S3** a-SMA expression in culured CFs stimulated by indicated concentrations of QL.
