# Supplementary figures and images for: Qiliqiangxin inhibits angiotensin II-induced transdifferentiation of rat cardiac fibroblasts through suppressing interleukin-6
Source: J Cell Mol Med. 2015 Mar 6;19(5):1114–21. doi: 10.1111/jcmm.12512 (PMC4420613; doi:10.1111/jcmm.12512)

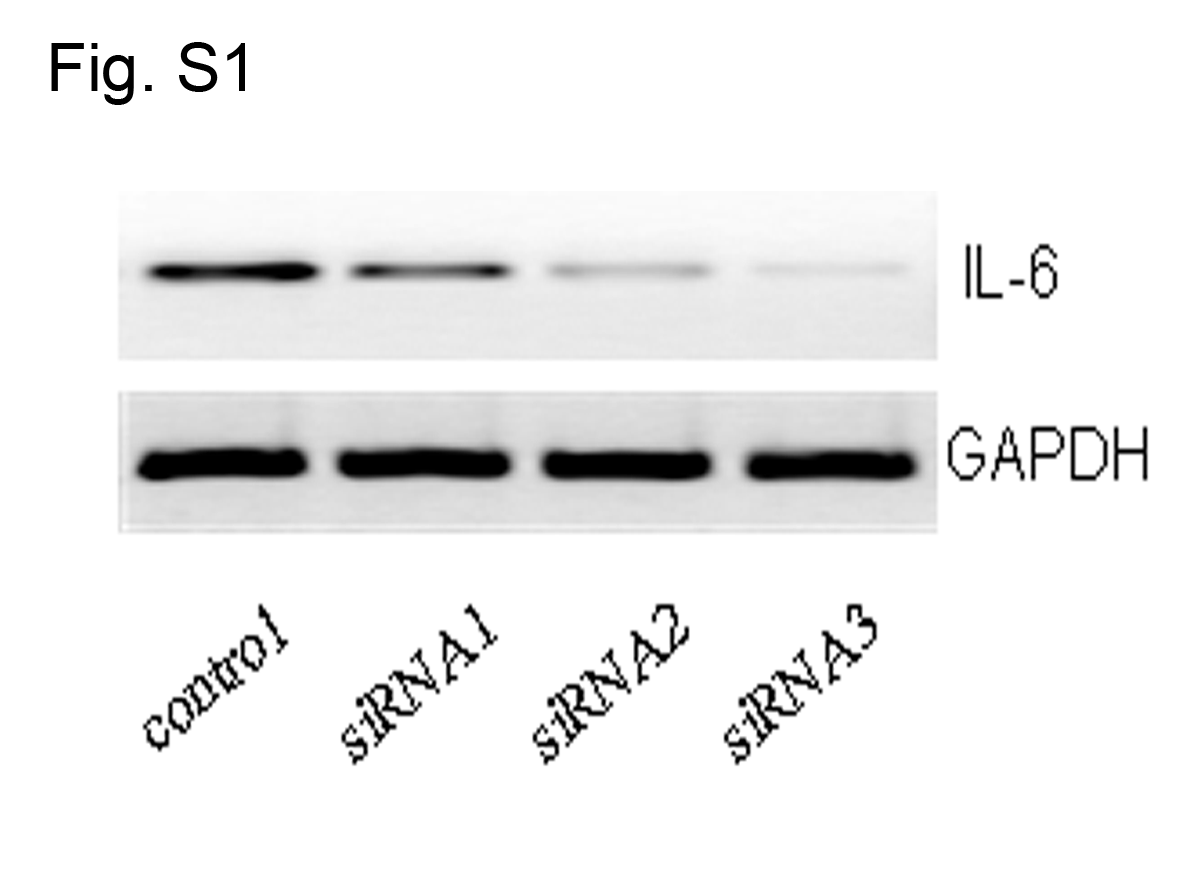

Supplement: Supplementary file 1 [file jcmm0019-1114-sd1.tif]

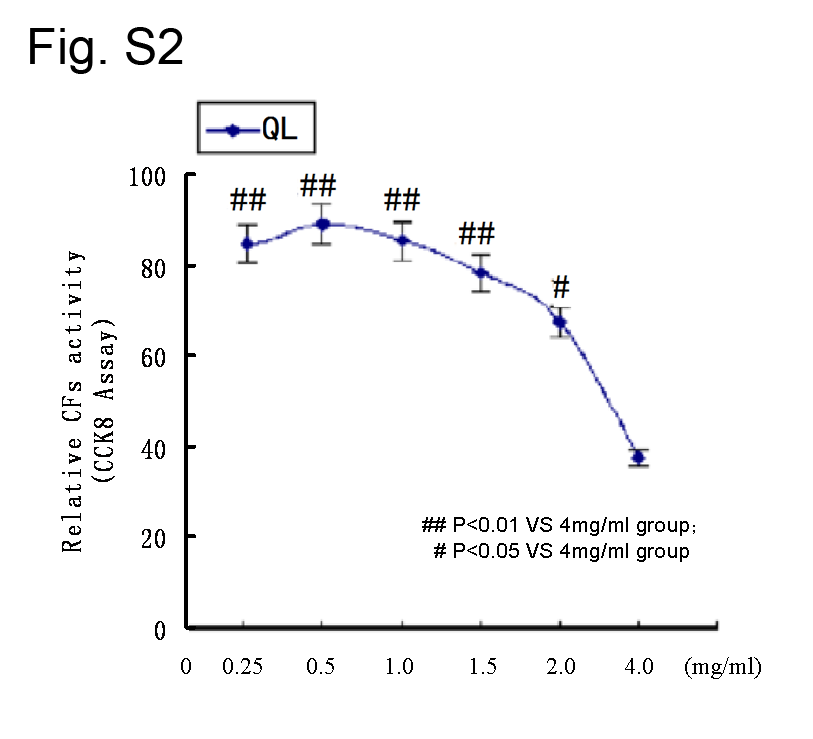

Supplement: Supplementary file 2 [file jcmm0019-1114-sd2.tif]

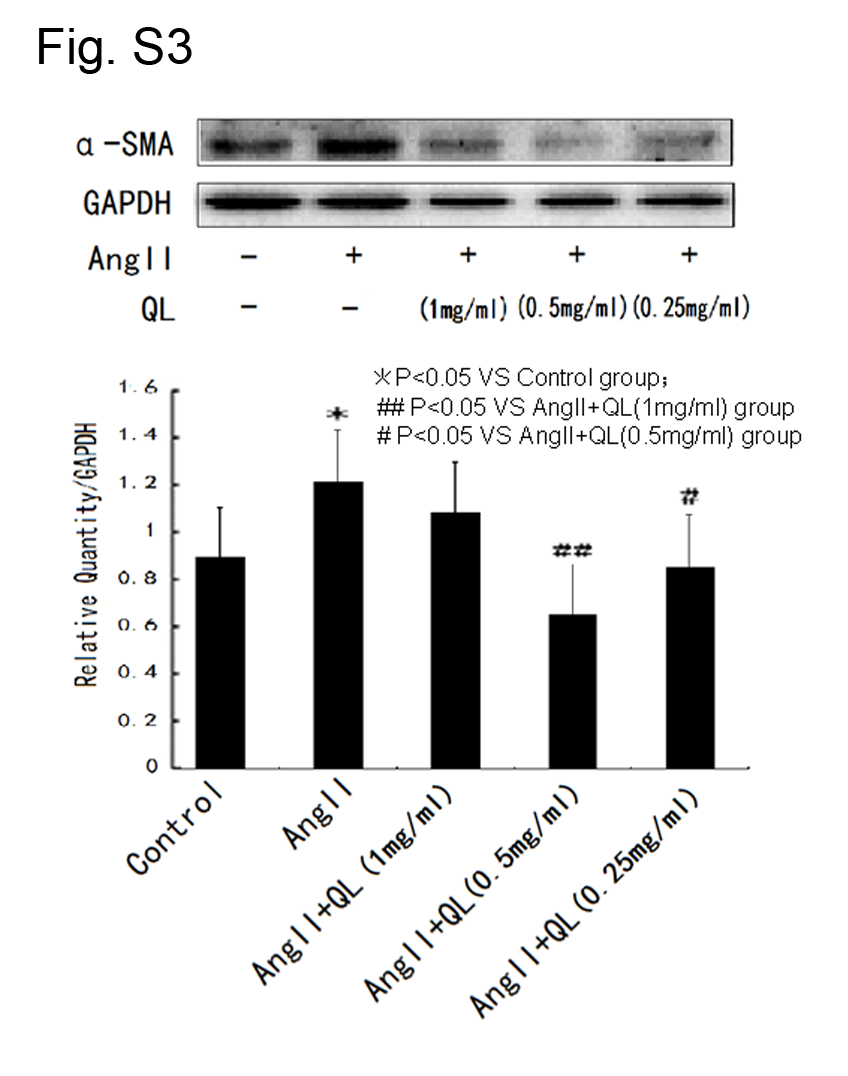

Supplement: Supplementary file 3 [file jcmm0019-1114-sd3.tif]
